# Input of Psychosocial Information During Multidisciplinary Team Meetings at Medical Oncology Departments: Protocol for an Observational Study

**DOI:** 10.2196/resprot.9239

**Published:** 2018-02-26

**Authors:** Melissa Horlait, Simon Van Belle, Mark Leys

**Affiliations:** ^1^ Organisation, Policy and Social Inequalities in Health Care Department of Health Sciences Vrije Universiteit Brussel Brussels Belgium; ^2^ Department of Medical Oncology Ghent University Hospital Ghent Belgium

**Keywords:** multidisciplinary collaboration, oncology, multidisciplinary communication, health services, qualitative research, multidisciplinary oncology consultations

## Abstract

**Background:**

Multidisciplinary team meetings (MDTMs) have become standard practice in oncology and gained the status of the key decision-making forum for cancer patient management. The current literature provides evidence that MDTMs are achieving their intended objectives but there are also indications to question the positive impact of MDTMs in oncology settings. For cancer management to be patient-centered, it is crucial that medical information as well as psychosocial aspects—such as the patients’ living situation, possible family problems, patients' mental state, and patients’ perceptions and values or preferences towards treatment or care—are considered and discussed during MDTMs. Previous studies demonstrate that failure to account for patients’ psychosocial information has a negative impact on the implementation of the treatment recommendations formulated during MDTMs. Few empirical studies have demonstrated the predominant role of physicians during MDTMs, leading to the phenomenon that medical information is shared almost exclusively at the expense of psychosocial information. However, more in-depth insight on the underlying reasons why MDTMs fail to take into account psychosocial information of cancer patients is needed.

**Objective:**

This paper presents a research protocol for a cross-sectional observational study that will focus on exploring the barriers to considering psychosocial information during MDTMs at medical oncology departments.

**Methods:**

This protocol encompasses a cross-sectional comparative case study of MDTMs at medical oncology departments in Flanders, Belgium. MDTMs from various oncology subspecialties at inpatient medical oncology departments in multiple hospitals (academic as well as general hospitals) are compared. The observations focus on the “multidisciplinary oncology consultation” (MOC), a formally regulated and financed type of MDTM in Belgian oncology since 2003. Data are collected through nonparticipant observations of MOC–meetings. Observational data are supplemented with semi-structured individual interviews with members of the MOC–meetings.

**Results:**

The protocol is part of a larger research project on communication and multidisciplinary collaboration in oncology departments. Results of this study will particularly focus on the input of psychosocial information during MDTMs.

**Conclusions:**

The concept of an MDTM should not merely be a group of care professionals who mostly work independently and occasionally liaise with one another. Interventions aiming to enhance the input of psychosocial information are crucial to ensure that MDTMs can benefit from their diverse membership to achieve their full potential. The findings from this study can be used to design nonclinical and organizational interventions that enhance multidisciplinary decision-making in oncology.

## Introduction

Multidisciplinary team meetings (MDTMs) have become standard practice in oncology and gained the status of the key decision-making forum for cancer patient management [1]. MDTMs aim for collaborative decision-making on treatment plans, ensuring that they are consistent with the best available evidence. MDTMs are considered to facilitate communication between healthcare professionals by gathering the relevant specialties around the table to share their knowledge and expertise and make collective evidence-based recommendations for patient management [2]. The current literature provides evidence that MDTMs are achieving intended objectives [3,4]. MDTMs lead to significant changes in the way cancer patients are assessed and managed [1,5] and lead to improved outcomes [6-8]. But there are also indications to question the positive impact of MDTMs in oncology settings [9].

The original Calman-Hine report (1995) recommends that “…cancer services should be patient centred and should take account of patients', families' and carers' views and preferences as well as those of professionals involved in cancer care. Individuals' perceptions of their needs may differ from those of the professional…” (page 6) [10]. According to this argumentation, it is crucial that medical, as well as nonmedical, information about the patient is discussed during MDTMs. Among the nonmedical information are psychosocial factors such as the patients’ living situation, possible family problems, patients' mental state, and patients’ perceptions and values or preferences towards treatment or care. These aspects help to put the results of the medical investigations and staging modalities in a broader perspective with a potential impact on the treatment plan and patient management [11]. Failure to account for patients’ psychosocial information has a negative impact on implementing treatment recommendations formulated within an MDTM [11,12].

Concerns arise over the lack of consideration for psychosocial information during multidisciplinary meetings [13-16]. Some individual studies demonstrated how the predominant role of physicians during MDTMs lead to medical information being shared, almost exclusively, at the expense of psychosocial information [13-16]. However, more in-depth insight on the underlying reasons why MDTMs fail to take into account psychosocial information of cancer patients is needed to enhance the effectiveness of the meetings given that MDTMs consume considerable time, effort, and financial resources [17-20].

This paper presents a research protocol for a cross-sectional comparative case study that will focus on the research question, “What are the personal, professional, organizational, and system-related barriers to consider psychosocial information during MDTMs at medical oncology departments?” This overall research question will be disentangled through subquestions addressing whether personal traits (gender, age, or experience) of MDTM participants facilitate or hamper the consideration of psychosocial information; whether professional background (medical, paramedical, psychological, nursing, etc.) of the participants affects positively or negatively the consideration of psychosocial information; whether department and organizational aspects (structural as well as cultural) affect the content and processes of a multidisciplinary dialogue; and to what extent health system characteristics (formal regulations, financing, and reimbursement rules) hamper or facilitate the uptake of psychosocial information during MDTMs. Moreover, as we want to develop a true understanding of the group dynamics during the MDTMs, we will address subquestions relating to how meeting habits, power plays and authority relationships, time constraints, and organizational values and norms potentially impact the consideration of certain information and the ultimate decision-making process. All these questions will be addressed on a case-by-case basis and with the additional research question: “What are the differences and commonalities between different MDTMs?”

## Methods

### Study Design

As part of a larger research project on communication and multidisciplinary collaboration in oncology departments [21], this protocol encompasses a cross-sectional comparative case study of MDTMs. A case study examines in rich detail the context and features of a social phenomenon within its context. The comparative part of the study aims at understanding and identifying general patterns and causal mechanisms over different contexts [22-25].

We use an interpretative research methodology grounded in the theoretical assumption that social realities (such as in MDTMs) are socially constructed through interaction and are very much context dependent. Emerging social patterns will be deduced from empirical fieldwork. The researchers aim for a better understanding of the determinants of the interaction processes and the factors influencing the participants’ experiences. This is done in an iterative process of reflection, data collection, data-ordering and triangulation, use of theories, and thus, interpretation. This also implies that no detailed or standardized rules are put forward to guide data acquisition; observations are guided by research questions and inductively emerging issues. Interviews are done in a semi-structured way offering variation in the framework for respondents. This qualitative research method aims at a holistic approach to capture the social reality of decision-making by combining induction and deduction to disentangle pathways of interdependent determinants for considering psychosocial information during MDTMs in oncological settings [26,27].

### Units of Analysis

MDTMs from various oncology subspecialties at inpatient medical oncology departments in multiple hospitals (academic as well as general hospitals) in Flanders (Belgium) are compared. MDTMs are defined as formally organized team meetings where medical (physicians) and nonmedical (nonphysicians) disciplines meet (whether physically in one place or by video- or teleconferencing) to discuss patient cases and to decide on treatment recommendations [21]. For this study, we focus on the “multidisciplinary oncology consultation” (MOC), a formally regulated and financed type of MDTM in Belgian oncology since 2003. It aims to foster multidisciplinary consultations within oncological settings and to ensure a systematic transparent multidisciplinary approach across all Belgian hospitals providing oncological programs [4,15,28]. Within the MOC, the multidisciplinary team should agree on the diagnosis of the patient and recommend a treatment plan based on clinical treatment guidelines.

An MOC is requested by the treating physician (general practitioner or specialist) and is legally mandatory in the following cases: a newly diagnosed cancer patient; when cancer treatment does not follow the accepted and written guidelines of the oncology department; when radiation therapy is repeated within one year; and when the administration of a new line chemotherapy is indicated. The MOC is a prerequisite for the reimbursement of certain chemotherapeutic treatments. The Belgian law states that the MOC must be chaired by a medical coordinator (preferably with oncological specialization). At least four different medical specialists (eg, radiotherapy, surgery, organ specialism, or pathology) from the hospital staff and one participant from outside the hospital (eg, the general practitioner or the treating physician of the patient if he/she is not part of the hospital team) participate [4,28].

In the daily hospital practice, MOCs (a legally required meeting per individual patient) are clustered in a collective meeting moment for all patients at stake, generally per tumour group, so called “MOC–meetings” [21]. The MOC–meetings are the units of analysis for this protocol.

### Data Collection

#### Nonparticipant Observations

Data are collected through nonparticipant observations of MOC–meetings. Nonparticipant observation is particularly useful as it allows the researchers to give an insider view about behavior, communication patterns, and other interactions between participants [29]. Data sources include audio recordings of the meetings and researchers’ field notes. Observations are guided by a supportive template of dimensions and issues to be considered. The template aims to organize descriptive data such as 1) frequency of the meeting, 2) duration, 3) composition and participation of disciplines, as well as more substantive data, such as 4) participants’ role during the meetings, 5) topics discussed during the meeting, and 6) the process of decision-making. The template can change when new dimensions emerge inductively from observations. Iteration in qualitative data collection and analysis is a reflexive process that involves continuous meaning-making, facilitating an in-depth understanding of the observed social reality.

#### Individual Interviews

Observational data are supplemented with semistructured individual interviews with members of the MOC–meetings. The interviews aim to increase understanding of the perspectives of the participants in the MOC–meetings.

We use interview guides with open-ended questions. All interviews start with an open-ended question to explore the respondents’ experiences of sharing psychosocial information during MOC-meetings. Subsequently, the interviews will focus on issues and dimensions emerging from the observations. The interviews aim at filling observation gaps, understanding how participants give meaning to emerging issues, and aim at respondent validation of the researchers' interpretations. The interviews will collect data on what the respondents perceive as barriers and facilitators to considering psychosocial information during the MOC–meetings. The individual interviews are recorded using a digital voice recorder.

All data will be anonymized and stripped of all sensitive personal and patient identifiers. Additional consent will be obtained for this data collection. Digital audio files are stored on a secured laptop and access to the data is only granted to the research team.

### Data Analysis

The individual interviews are transcribed. Transcripts as well as the researchers’ field notes and written comments from the observations are used in the analysis process. Two researchers independently code the data thematically and then discuss and compare emerging categories, subcategories, and interpretations of the findings. A third researcher will be consulted in case of disagreement. A preliminary thematic analysis is performed after each observation. Initial content issues or identified patterns are used to support subsequent observations for targeted topics to expand upon or validate hypotheses (the iterative cycle of qualitative research) [30].

## Results

The protocol is part of a larger research project on communication and multidisciplinary collaboration in oncology departments which is funded by The Research Foundation – Flanders (G035813N). Preliminary results on MDTMS in oncology settings have been published previously [15]. Results of this study will particularly focus on the input of psychosocial information during MDTMs.

### Ethics Approval

Ethics approval for this study was given by the central Medical Ethics Commission of the Brussels University Hospital (BUN 143201318799). Additional approval will be obtained from the participating organizations.

## Discussion

Qualitative research focusing on multidisciplinary collaboration and organizational issues within the health system that determine the content and work processes of MDTMs is rather scarce. Within oncology, most studies on MDTMs are quantitative and focus on the impact of MDTMs on patient assessment, management, and outcomes [1]. This qualitative cross comparative case study approach aims to contextualize variables which cannot be captured with quantitative research methods due to the complexity and circumstantial factors affecting interactions. This research strategy aims to get a true insider-view on the MOCs and to capture the experiences and meanings of the professionals involved. It aims to reveal patterns of the professional interaction in multidisciplinary team meetings and how different professionals contribute, use, and assess psychosocial information in the process of collective decision making. Moreover, the study aims to better understand barriers and facilitators in the practice of oncology to considering psychosocial information during MDTMs.

This type of research has the potential for practical value as MDTMs are costly in terms of both time and money [17-20]. Major concerns arise surrounding the practicality of integrating “overhead tasks,“ such as MDTMs, into the heavily loaded work schedules of many professionals. There is a need to explore how MDTM practices can be integrated efficiently considering health care professionals' limited time. Determinants of organizational and health system decisions could potentially explain why MDTMs do not incorporate psychosocial factors [21].

The concept of a MDTM should not merely be a group of care professionals who work essentially independently and occasionally liaise with one another. One of the main goals is to develop recommendations for integrating psychosocial information that are empirically grounded in an understanding of the patterns of multidisciplinary dialogue and decision making in oncology in various types of hospitals. This research protocol will be the first step to enabling future international comparative studies that take into account health system characteristics. The findings from this study can also be used to design nonclinical and organizational interventions that enhance multidisciplinary decision-making in the context of specific teams, organizations, and health systems. Interventions aiming to enhance the input of psychosocial information are crucial to ensure that MDTM can benefit from their diverse membership to achieve their full potential.

## Abbreviations

MDTM: multidisciplinary team meeting
MOC: multidisciplinary oncology consultation
